# Preparation of FA-targeted magnetic nanocomposites co-loading TFPI-2 plasmid and cis-platinum and its targeted therapy effects on nasopharyngeal carcinoma

**DOI:** 10.7150/ijms.52643

**Published:** 2021-04-09

**Authors:** Fang Liu, Bojie Chen, Weifeng Chen, Shuaijun Chen, Dong Ma, Minqiang Xie

**Affiliations:** 1 Department of Otorhinolaryngology Head and Neck Surgery, First Affiliated Hospital of Gannan Medical University, Ganzhou, 341000, China.; 2 Department of Joint Surgery, The Affiliated Ganzhou Hospital of Nanchang University, Ganzhou, 341000, China.; 3 Department of Otolaryngology, Zhujiang Hospital, Southern Medical University, Guangzhou 510282, China.; 4 Key Laboratory of Biomaterials of Guangdong Higher Education Institutes, Department of Biomedical Engineering,Jinan University, Guangzhou, 510632, China.

**Keywords:** magnetic nanoparticles, folate acid, TFPI-2, cisplatin, nasopharyngeal carcinoma, targeted delivery

## Abstract

The majority of patients diagnosed with nasopharyngeal carcinoma (NPC) present with advanced-stage disease. The main treatment for these patients is concurrent chemoradiotherapy, which has various side effects. To improve the therapeutic effects and reduce the side effects of NPC chemoradiotherapy, we constructed a multifunctional folic acid (FA)-targeted magnetic nanocomposite codelivering tissue factor pathway inhibitor-2 (TFPI-2) and cisplatin (CDDP). This novel nanocomposite (FA-MNP/CDDP/TFPI-2) was obtained by amidation and electrostatic adsorption between FA-methoxypolyethylene glycol-polyethyleneimine (FA-MPEG-PEI) containing the TFPI-2 plasmid and magnetic nanoparticles modified by aldehyde sodium alginate loaded with CDDP. Transmission electron microscopy (TEM) images showed that the size of the individual magnetite particle core was approximately 11.5 nm. The structure and composition of the nanocomposites were identified and examined by ^1^H nuclear magnetic resonance (NMR) spectroscopy and ultraviolet (UV) spectrophotometry. The fluorescence analysis, Prussian blue iron staining, magnetic resonance (MR) imaging and whole-body fluorescence imaging results demonstrated that FA-MNP/CDDP/TFPI-2 showed high gene transfection efficiency and could target tumor cells via folate receptor (FR)-mediated delivery. The codelivery analysis showed that the obtained FA-MNP/CDDP/TFPI-2 composite could cause significantly more apoptosis than treatment with CDDP or TFPI-2 alone. The results showed that the FA-MNP/CDDP/TFPI-2 composites were successfully synthesized and indicated to be a specific molecular target for the FR with significant inhibitory effects on the growth of HNE-1 cells.

## Introduction

Nasopharyngeal carcinoma (NPC) is a particularly prevalent cancer in southern China and southeast Asia. Although intensity-modulated radiotherapy (IMRT) provides excellent local-regional control for advanced NPC, the average five-year survival rate of advanced NPC is still undesirable 1-3. In recent years, chemoradiotherapy has received increasing attention in the field of NPC treatment research 4. Magnetic nanoparticles (MNPs) have drawn substantial interest and have been widely used in various biomedical applications, including targeted drug delivery and hyperthermia-based therapy, due to their unique properties such as biocompatibility and biodegradability as well as magnetic and heat-mediated characteristics 5, 6. Our team has previously studied magnetic nanocomposites in targeted therapy for nasopharyngeal carcinoma and demonstrated that magnetic nanocomposites have good biocompatibility 7, 8.

The anticancer drug cisplatin (CDDP) has been used generally in the treatment of solid tumors, such as prostatic cancer 9, NPC 10, lung cancers, and some successes 11 have been achieved. However, the applications of CDDP are limited due to its poor aqueous solubility and serious adverse effects associated with nephrotoxicity myelosuppression and gastrointestinal adverse reactions 12. Furthermore, the nonspecific systemic distribution of CDDP commonly gives rise to dose-dependent toxic effects and poor therapeutic outcomes 13. To resolve these problem, biodegradable magnetic nanoparticles (MNPs) functioning with methoxypolyethyleneglycols (MPEG) on the surface have been successfully applied to enclose CDDP, leading to its improved water solubility and bioavailability, and fewer CDDP-associated side effects and better therapeutic benefits 14.

Tissue factor pathway inhibitor-2 (TFPI-2) can inhibit various members of the matrix metalloproteinase (MMP) family of photolytic enzymes. Additionally, TFPI-2 has been implicated in maintaining the integrity of the structure of the extracellular matrix (ECM) structure, and inhibiting the invasion and metastasis of tumor cells. Reduced TFPI-2 expression in pancreatic cancer 15 cervical cancer 16 and NPC 17 can consequently promote tumor invasion and metastasis. TFPI-2 can also significantly inhibit the invasion and metastasis of melanoma 18 and lung cancer cells 19,20. Considering these data, we postulate that TFPI-2 gene therapy can inhibit the growth of some tumors. Gene therapy, gene carriers play a vital role in gene therapy. As a gene carriers, liptosomes exhibit high toxicity 21, and adenoviruses present with a high risk of virus activation and a lack of specific targeting 22. Therefore, a nonviral vector is an attractive gene carrier option, although the current agents being used display disadvantages, such as low transfection efficiency and high toxicity 23.

In the recent years, molecular targeted therapy has been the major strategy in cancer research, because it can enhance the therapeutic effects, reduce side effects, prevent drug resistance, and so on 24,25. To overcome the gene carrier problem of high cationic toxicity (e.g., polyethyleneimine (PEI)) and low transfection efficiency (e.g., polyethylene glycol (PEG)-PEI), our team linked the cell-targeting molecule folic acid (FA) onto PEG and PEI 26. FA is a targeting ligand used for anticancer agents, since its target, the folate receptor (FR), is often overexpressed in tumor cells (e.g., the HNE-1 cell line) and rarely found in normal tissue 27. Therefore, the FA has been used to test the enhancement effects on vector delivery in FR-positive tumor cells 28. In this study, we established a FA-targeted drug that delivers TFPI-2 and CDDP (FA-MNP/CDDP/TFPI-2) for NPC therapy. In this complex, MNPs function as the carrier, FA functions as the targeting moiety, TFPI-2 is included for gene therapy, and CDDP is the antitumor drug. The FA-targeting properties and antitumor effects of FA-MNP/CDDP/TFPI-2 were investigated *in vitro* and *in vivo*.

## Methods

### Materials

MPEG (2 kDa), PE (25 kDa), FA, CDDP, N,N'-dicyclohexylcarbodiimide (DCC) and N-hydroxysuccinimide (NHS) were purchased from Sigma (USA) and used directly. The pEGFP-C1 plasmid, which was used for construction of the vectors expressing TFPI-2, were from GenePharma Co., Ltd. (Suzhou, China). Roswell Park Memorial Institute (RPMI) 1640 medium, fetal bovine serum (FBS), and Dulbecco's phosphate-buffered saline (PBS) were purchased from Gibco (Life Technologies) Corporation (USA). The Prussian blue dye solution method (neutral red) kit was purchased from Beijing Leagene Biotech Co., Ltd. Cell Counting Kit-8 (CCK-8) was purchased from Dojindo Laboratories (Kumamoto, Japan). Annexin V FITC-A and cell cycle kits were purchased from Becton Dickinson and Company (USA). The TFPI-2 antibody was purchased from Santa Cruz (USA). RNAiso Plus was purchased from Takara (Japan). Human NPC HNE-1 and CNE-2 cells were supplied by the Central Laboratory of Central South University.

### Synthesis of FA-MNP/CDDP/TFPI-2

The MNPs were first prepared using a chemical precipitation technique. Briefly, FeCl_3_·6H_2_O (2.70 g) and FeSO_4_·7H_2_O (1.39 g) were codissolved in 100 mL of distilled water and added to 10 mL of NH_3_·H_2_O with vigorous stirring. The Fe_3_O_4_ products were exhaustively washed with the help of a strong magnet. Sodium alginate (SA) (4 g) and sodium periodate (1 g) were codissolved in 100 mL of distilled water. After the reaction, aldehyde sodium alginate (ASA) was ultrafiltered (MWCO=2000) to detach the unreacted micromolecules. Then, the Fe_3_O_4_ products and ASA were mixed and stirred in an 85 °C water bath for 3 min. After centrifugation and removal of the precipitate, the MNP products were ultrafiltered (MWCO=1000) to detach SA without the aldehyde. CDDP (10 mg) was then dissolved in distilled water and added to MNPs (3 ml). The mixture was shaken at 37 °C for 24 h and dialyzed (MWCO=14000) for 72 h to detach the dissociated CDDP, thereby synthesized MNP-ASA-CDDP. Since ASA is a surface modifier and has no substantial effect on treatment, we usually abbreviate this mixture as MNP-CDDP.

For the preparation of MPEG-COOH, 10 g of MPEG (5 mmol) and 2.5 g of succinic anhydride (25 mmol) were codissolved in 50 mL of dried N,N-dimethylformamide (DMF). The solution was stirred under a nitrogen atmosphere for 8 h and then cooled to 0 °C. After the reaction, the product was dialyzed in distilled water for 3 days (MWCO=500) and lyophilized, resulting in a new conjugate (MPEG-COOH). To confirm the click reaction, samples were taken before and after conjugation for Fourier transform infrared FTIR analyse 29. FTIR spectra were recorded on a Perkin-Elmer Paragon 1000 spectrometer at frequencies ranging from 500 to 4000 cm^-1^. For the preparation of FA-MPEG-PEI, 5 g (0.04 mmol) PEI, 1.0 g (0.5 mmol) MPEG-COOH and 1.0 g (2.0 mmol) of FA were dissolved in 30 mL of dried dimethyl sulfoxide (DMSO). Then, 0.6 g (4.8 mmol) DCC and 0.35 g (3 mmol) NHS were added to the solution. The system was stirred at room temperature for 24 h. The product was dialyzed in distilled water for 3 days (MWCO = 8000) and lyophilized, resulting in a new conjugate (FA-MPEG-PEI). To confirm the click reaction, samples were taken for ^l^H nuclear magnetic resonance (NMR) analysis. The ^1^H NMR spectra were obtained in D_2_O by using a Bruker DPX-300 NMR spectrometer (300 MHz) at room temperature.

The FA-MPEG-PEI conjugate was first dissolved in ultrapure water at room temperature and then mixed with the TFPI-2 plasmid at an appropriate concentration. After the reaction, the product was stirred with MNP-CDDP at an appropriate mass ratio for the formation of the FA-MNP/CDDP/TFPI-2 complexes. No signs of particle precipitation were observed over a period of at least 8 months.

### Characterization of FA-MNP/CDDP/TFPI-2

To determine the loaded amount of CDDP, the FA-MNP/CDDP/TFPI-2 complexes were dissolved in 6 mL of 12-oxophytodienoic acid (OPDA)/DMF (1.2 mg/mL) and measured at 1 nm by ultraviolet (UV) spectrophotometry^30^ (Encapsulation rate%= CDDP content /CDDP integral dose×100%). The benzene ring structure of the FA molecule has characteristic absorption peaks at 256 nm, 283 nm, and 365 nm. With these features, we adopted UV spectrophotometry (UVIKON923 BIO-TEK, USA) to analyze the content of FA in the complexes 31. The ^1^H NMR spectrum of FA-MPEG-PEI was obtained on a Gemini-200 spectrometer (Varian, CA, USA) using D_2_O as the solvent to detect the chemical bonds of FA-MPEG-PEI.

Morphological examination of the complexes was performed using a JEM-2010HR high-resolution transmission electron microscopy (TEM) after counterstaining with uranyl acetate. For the resulting complexes, their particle sizes and zeta potentials were determined with a zeta potential analyzer (ZetaPALS, Brookhaven Instruments Corporation, USA).

### Gel electrophoresis

The ability of FA-MNP/CDDP to bind to the TFPI-2 plasmid was examined by agarose gel electrophoresis (AGE). Agarose gel (1.0%) containing ethidium bromide (EB) (0.25 mg/mL, Sigma) was prepared in TAE buffer (40 mmol/L Tris acetate, 2 mmol/L EDTA). All samples were separated by electrophoresis on an agarose gel at 60 V for 1 h. Visualization and image capture were accomplished using a UV transilluminator with the Kodak EDAS 290 digital imaging suite (Fisher Scientific, PA).

### *In vitro* transfection

FR-positive human NPC HNE-1 cells and FR-negative human NPC CNE-2 cells were used to study the FA-targeting function of FA-MNP/CDDP/TFPI-2. HNE-1 cells and CNE-2 cells were cultured at a density of 1×10^4^ cells per well in 6-well tissue culture plates in no-FA RPMI 1640 medium and then incubated in a humidified 5% CO_2_ atmosphere at 37 °C for 24 h. After that, freshly prepared FA-MNP/CDDP/TFPI-2 with serum-free no-FA RPMI 1640 was added.

The amount of the TFPI-2 plasmid in each well was fixed at 5.0 µg for incubation in a humidified 5% CO_2_ atmosphere at 37 °C. After 5 h of incubation, the formulations were removed, 2 mL of fresh RPMI 1640 culture medium was added followed by culture for an additional 48 h. The cells were then analyzed for green fluorescent protein (GFP) expression with a fluorescence microscope (Nikon-2000U, Japan).

The concentration of Fe in each well was fixed at 10 μg/mL for incubation in a humidified 5% CO_2_ atmosphere at 37 °C for 3 h. The formulations were removed and the cells were washed with PBS. Prussian blue stain was added to each well for 30 min, and neutral red dye solution was added for 10 s after fixation with 4% paraformaldehyde for 10 min. The cells were observed by inverted microscopy (Zeiss, Germany), and each test was repeated at least three times.

### mRNA and protein expression

HNE-1 and CNE-2 cells (5×10^4^) were seeded in 6-well plates and incubated at 37 °C in 5% CO_2_ for 24 h. Various formulations (TFPI-2 and FA-MNP/CDDP/TFPI-2) were added followed by incubation with the cells for 48 h for mRNA isolation and protein extraction. The cellular levels of TFPI-2 mRNA and protein were evaluated by RT-PCR and Western blot, respectively.

For RT-PCR analysis, total RNA was extracted from treated cells using TRIzol reagent (Invitrogen). The integrity of the RNA was examined with a microspectrophotometer. Then, 1.5 µg of total RNA was transcribed into cDNA using the reverse transcriptase M-MLV (RNase H). Thereafter, 5 µl of cDNA was subjected to RT-PCR analysis targeting TFPI-2 and β-actin using *Premix Ex Taq*™ (Tli RNaseH Plus) (Takara, Japan). The PCR parameters consisted of 45 cycles (denaturation at 95 °C for 30 s, annealing at 60 °C for 30 s, and elongation at 72 °C for 30 s) followed by analysis with ViiA7 software (ABI, USA). The expression of miRNA was normalized to the expression of the endogenous control, 18S rRNA. Relative gene expression values were determined using Quality One Software. The primers used in RT-PCR for TFPI-2 and β-actin were as follows:

- TFPI-2 F: 5′-CTT CTC CGT TAC TAC TAC GAC AGG T-3′,
- R: 5′-GCC TCC CAG GTG TAG AAA TTG TTG-3′;
- β-actin F: 5′-CCT GGA TAC CGC AGC TAG GA-3′,
- R: 5′-GCG GCG CAA TAC GAA TGC CCC-3′.

For Western blot analysis, the transfected cells were washed twice with cold PBS and lysed with lysis buffer (50 mM Tris HCl, pH=7.4, 150 mM NaCl, 1% Triton X-100, 10% glycerol, 1.5 mM MgCl_2_, 1 mM EDTA) (Santa Cruz). The cell lysates were incubated on ice for 30 min with vortexing every 5 min. The lysates were then clarified by centrifugation for 10 min at 12000 r/min. The supernatant was boiled in loading buffer for 10 min. Total protein (20 mL) was separated on 12% PAGE-SDS gels and then transferred to PVDF membranes (Bio-Rad). After incubation in phosphate-buffered saline with Tween-20 (PBST, pH 7.2) containing 5% bovine serum albumin (BSA) (Merck, Germany) for 1 h, the membranes were incubated in PBST containing 5% BSA with TFPI-2 antibodies (1:2000) overnight. After incubation in PBST containing 5% BSA with goat anti-rabbit IgG-HRP antibody (1:4000) for 60 min, the bands were visualized using an ECL system (Pierce). Relative gene expression values were determined using ImageJ software and β-actin were used as a reference.

### Cellular toxicity

A Cell Counting Kit-8 (CCK-8) assay was used to evaluate the cytotoxicity of FA-MNP/CDDP/TFPI-2. HNE-1 cells were cultured in a 96-well plate (1×10^4^ cells/well) in complete RPMI 1640 culture medium supplemented with 10% FBS in a humidified atmosphere of 5% CO_2_ at 37 °C for 24 h. The growth medium was replaced with 100 µl of complete no-FA RPMI 1640 culture medium containing the desired amount of FA-MNP, MNP-CDDP, FA-TFPI-2, or FA-MNP/CDDP/TFPI-2 (CDDP concentration of 1.5ug/ml and TFPI-2 concentration of 2.5 µg/ml), and five wells were set for each sample. Cells treated with PBS were used as the control group. The cells were incubated for another 24 h, and 10 µl of CCK-8 solution was added to every well. After incubation at 37 °C for another 2 h, the absorbance that correlated with the number of viable cells in each well was measured with an absorbance microplate reader at a wavelength of 450 nm.

Inhibition Ratio* (%) =1-[A(experimental)-A(blank control)]/[A(negative control)-A(blank control)] ×100%

### Apoptosis assay

HNE-1 cells seeded on 6-well plates were treated with FA-MNP, MNP-CDDP, FA-TFPI-2, or FA-MNP/CDDP/TFPI-2 (CDDP concentration of 1.5 µg/ml and TFPI-2 concentration of 2.5 µg/ml) at 37 °C for 48 h. Then, all cells were trypsinized, collected and washed with ice-cold PBS three times. The cells were resuspended in 200 µl of binding buffer. Thereafter, 5 mL of Annexin V APC-A and 10 µl of propidium iodide (PI) were added followed by mixing for 15 min in the dark. The stained cells were analyzed using a flow cytometer.

### Cell cycle analysis

HNE-1 cells seeded on 6-well plates were treated with FA-MNP, MNP-CDDP, FA-TFPI-2, or FA-MNP/CDDP/TFPI-2 (CDDP concentration of 1.5 µg/ml and TFPI-2 concentration of 2.5 µg/ml) at 37 °C for 48 h. At the end of incubation, the cells were added to ice-cold 70% ethanol and stored at -20 °C for 12 h. After centrifugation at 2000 rpm for 3 min at 4 °C and washing with PBS twice, the cells were resuspended in 500 µl of PBS buffer containing DNase-free RNase A and PI. After 30 min of nuclear staining, all samples were analyzed by Beckman Coulter Cytomics FC-500 and Kaluza flow cytometry software. The resulting cell cycle was analyzed for the proportion of cells undergoing apoptosis and those in the G1, G2, S, and G2/G1 phases of the cell cycle.

### *In vivo* targeting

Female 5-week-old BALB/c nude mice (18 g) were purchased from Sun Yat-sen University Animal Laboratory Center (Guangzhou, China). All animal experiments were performed in agreement with the guidelines of the Institutional Committee for Animal Care and in accordance with the policy of the National Ministry of Health.

#### *In vivo* magnetic resonance imaging (MRI) of the MNPs in tumor-bearing mice

To establish tumor models, approximately 1×10^7^ HNE-1 cells/mouse and CNE-2 cells/mouse were subcutaneously implanted into the left and right oxters of each nude mouse, respectively. After the xenografted tumor nodules had grown for 3 weeks, a solution of FA-MNP/CDDP/TFPI-2 in PBS (0.2 mL, 0.5 mg of Fe per mouse) was intravenously delivered to the mice via the tail vein. MR scanning of the mice was performed using an Achieva 3.0T superconductor clinical MR system (Philips, Holland) at 24 h postinjection. After anesthetization by intraperitoneal injection of pentobarbital sodium (50 mg/kg), both the HNE-1 tumor and CNE-2 tumor-bearing mice were placed inside a custom-built rodent receiver coil, and 2D MR images were obtained.

#### *In vivo* fluorescence imaging of GFP in tumor-bearing mice

To establish the tumor models, approximately 1×10^7^ HNE-1 cells and approximately 1×10^6^ CNE-2 cells were subcutaneously implanted into the left oxters of nude mice. After the xenografted tumor nodules had grown for 3 weeks, a solution of FA-MNP/CDDP/TFPI-2 in PBS (0.2 mL, 0.05 mg of TFPI-2 plasmid per mouse) was intravenously delivered to the mice via the tail vein. After anesthetization, the tumor-bearing mice were positioned on a glass platen at a fixed distance from the excitation source and imaged using a Caliper IVIS Lumina II at 48 h postinjection. *In vivo* spectral imaging from 465 nm to 535 nm was carried out with an exposure time of 10 s for each image frame.

#### *In vivo* toxicity and apoptosis assay

At 14, 16, 18, 20, and 22 days after implantation, FA-MNP, MNP-CDDP, FA-TFPI-2, or FA-MNP/CDDP/TFPI-2 (CDDP, 3 mg/kg; TFPI-2 plasmid, 2 mg/kg) was intravenously delivered to the mice via the tail vein at a dose of 200 µl per mouse. On the 28th day, the mice were anesthetized with diethyl ether and sacrificed by decapitation. The tumors were removed and fixed in 4% paraformaldehyde for 24 h. Detection of apoptotic cells was performed with the TUNEL method using an *in situ* apoptosis detection kit. Sections were incubated with proteinase K for 15 min at room temperature, and endogenous peroxidase was blocked with a solution of PBS and 3% H_2_O_2_ for 10 min. Detection of the antigen-antibody link was performed with immunoperoxidase followed by diaminobenzidine (DAB) chromogenesis. The sections were counterstained with hematoxylin, rinsed with distilled water and observed by microscopy.

### Statistical analysis

Data were processed using Microsoft Excel 2007 software and are presented as the mean±standard error of the mean (S.E.M.). Statistical analyses were performed employing the one-tailed Student's t-test using the statistical software SPSS 13. The level of significance was set at *p*<0.05. All graphs were plotted by Microsoft Office PowerPoint Adobe Illustrator CS6 and Adobe Photoshop CS5.

## Results and Discussion

### Characterization of FA-MNP/CDDP/TFPI-2

**Figure 1:**
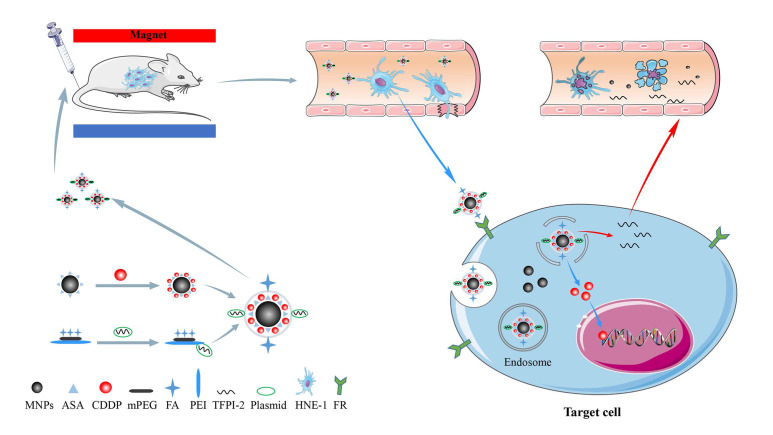
Schematic illustration for the synthesis of FA-MNP-CDDP/TFPI-2 and chemoradiotherapy of nasopharyngeal carcinoma HNE-1 in nude mouse through intravenous injection. **Abbreviations:** MNPs, Magnetic nanoparticles; ASA, aldehyde sodium alginate; CDDP, cisplatin; mPEG, Methoxypolyethyleneglycols; FA, folic acid; PEI, polyethyleneimine; TFPI-2, tissue factor pathway inhibitor 2.

**Figure 2:**
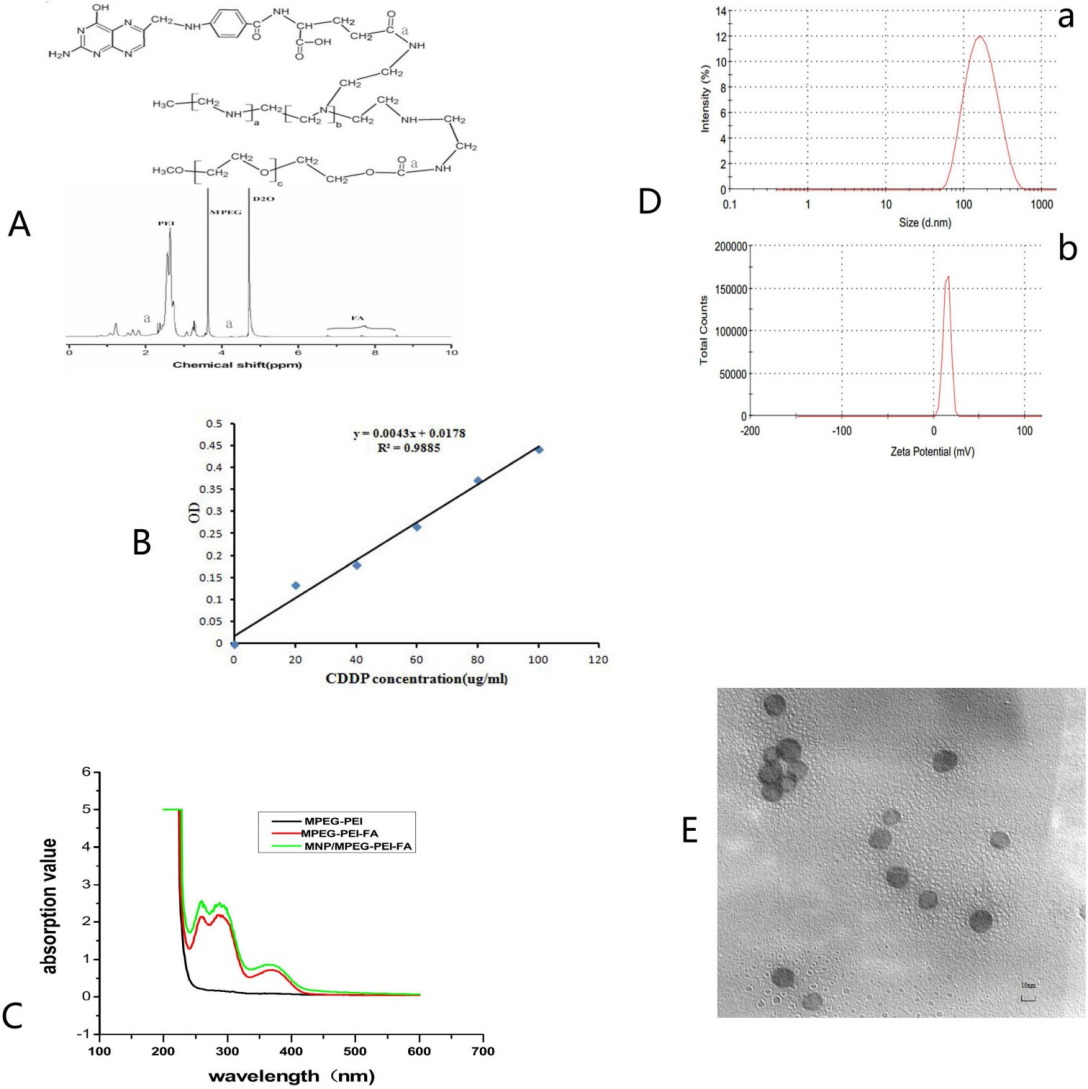
** (A)** Chemical structure and 1 H NMR spectrum of FA-MPEG-PEI. **(B)** The standard curve of CDDP. **(C)** UV spectrum of MPEG-PEI, FA-MPEG-PEI, MNP/ FA-MPEG-PEI. **(D)** Particle sizes and zeta potentials of FA-MNP/CDDP/TFPI-2. **(E)** TEM image of the FA-MNP/CDDP/TFPI-2.

Our previous work synthesized the PEG-PEI(FA)-Alg-Fe_3_O_4_^26^ and MNP-CDDP^32^.On this basis, we synthesized water-dispersible FA-MNP/CDDP/TFPI-2 with superparamagnetic iron oxide nanoparticles. The whole process of FA-MNP/CDDP/TFPI-2 and its space diagram is shown in the lower left corner of Figure 1. FA-MNP/CDDP/TFPI-2 was tested *in vivo* and *in vitro* in nude mice and the schematic diagram is shown in right of Figure 1. The magnetic nanocomposite specifically binds to the folate receptors tumor cells, and releases the drug cisplatin and the gene TFPI-2 in tumor cells, which help kill tumor cells. Figure 2A shows the chemical structure and^ 1^H NMR spectrum of FA-MPEG-PEI, and all signals are marked. For FA, the characteristic peaks appeared between 6.68-8.68 ppm 33, the characteristic peaks of D_2_O groups appeared at 4.79 ppm 33, the characteristic peaks of the MPEG group appeared at 3.65 ppm 34, and the characteristic peaks of the PEI group appeared at 2.8 ppm 35. After the click conjugation, the obtained FA-MPEG-PEI showed not only the characteristic peaks of FA, MPEG and PEI but also new peaks at 2.1, 4.2, 4.6, 7 and 8.17 ppm, which could be attributed to the presence of the amide due to the dehydration condensation reaction 36.

OPDA was used to determine the content of CDDP, and its standard curve is shown in Figure 2B (*y*=0.0043*x*+0.0178 R2=0.9885). The OD of UV in 703 nm was 0.673±0.028.We calculated the average drug loading of CDDP is 150 ug/mL, and the encapsulation rate was 37.25%.

The ultraviolet spectrum was analyzed to qualitatively determine the FA on FA-MNP/CDDP/TFPI-2. As shown in Figure 2C, the profound UV absorbance peaks at approximately 256, 283 and 283 nm can be attributed to the aromatic ring of FA^37^,^38^.

### Morphology, sizes and zeta potentials of the FA-MNP/CDDP/TFPI-2 complexes

The particle sizes of the FA-MNP/CDDP/TFPI-2 complexes ranged from 50 nm to 400 nm, with an average size of 151 nm. The zeta potentials ranged from 0 mV to 28.2 mV, with an average of 15.4 mV (Figure 2D). Moreover, TEM observations were used to characterize complex formation (Figure 2E). The particle sizes of FA-MNP/CDDP/TFPI-2 complexes ranged from 9.8 nm to 13.3 nm, and they showed good monodispersity with a spherical shape.

### Gel electrophoresis

**Figure 3:**
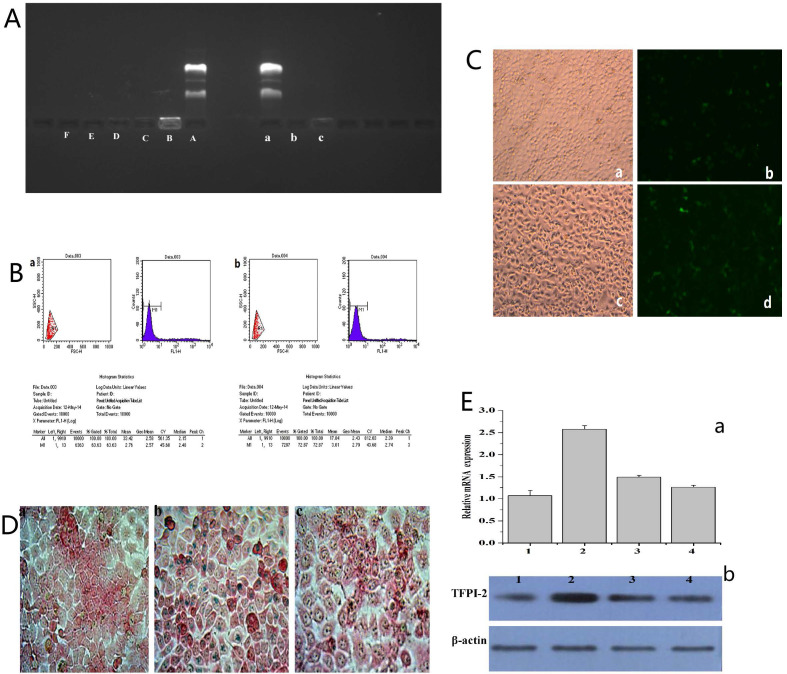
** (A)** (A~F) Agarose gel electrophoresis retardation assay of FA-MNP/CDDP complexesat various N/P ratios; (a~c) DNase I degradation assay for FA-MNP/CDDP complexes at various N/P ratios. **(B)** Quantitative determination of transfected CNE-2 (a) and HNE-1 (b) cells by flow cytometry. **(C)** Fluorescence microscopy of green fluorescent protein expression. a: CNE-2 Optical microscope figure; b: CNE-2 Fluorescence microscope figure; c: HNE-1 Optical microscope figure; d: HNE-1 Fluorescence microscope figure (100×). **(D)** Iron stain: a: CNE-2; b: HNE-1; c: HNE-1 close folate receptor (400×). **(E)** (a) Expression of TFPI-2 mRNA determined by RT-PCR. (b) Representative TFPI-2protein expression determined by Western blot analysis (1: HNE-1 TFPI-2; 2: HNE-1 FA-MNP/CDDP/TFPI-2; 3: CNE-2 FA-MNP/CDDP/TFPI-2; 4: CNE-2 TFPI-2).

The ability of FA-MNP/CDDP to bind to the TFPI-2 plasmid was examined by gel electrophoresis. As shown in Figure 3A(A~F), FA-MNP/CDDP could not entirely bind to the TFPI-2 plasmid, and migratory TFPI-2 could be observed at low nitrogen/phosphate (N/P) ratios (>10). When the N/P ratio was equal to or higher than 10, FA-MNP/CDDP completely retarded the electrophoretic mobility of TFPI-2. The protective effects of the complexes against DNA degradation by DNase I is shown in Figure 3A(a~c). The naked TFPI-2 plasmid was completely digested, whereas FA-MNP/CDDP/TFPI-2 exhibited distinct protective effects against DNase I at both N/P ratios (10, 20) 39. These results indicated that the MNPs could not only load hydrophobic drugs but also bind and protect genes against DNaseI. Hence, these particles showed potential applications in drug and gene codelivery.

### Cell culture

#### Gene transfection efficiency

To explore the possibility of using FA-MNP/CDDP for gene delivery, we preliminarily investigated the transfection of FA-MNP/CDDP/TFPI-2 complexes by using CNE-2 and HNE-1 cells. The transfection efficiencies was measured using flow cytometry, which CNE-2 group is 63.63% and HNE-1 is 72.87% (Figure 3B), and Figure 3C shows the fluorescence images of the transfected HNE-1 and CNE-2 cells. FR-positive HNE-1 cells exhibited the high transfection efficiency of 37.4%, which was significantly higher than that of the FR-negative CNE-2 cells at 27.2%. This result indicated that FA-MNP/CDDP/TFPI-2 can target FR-positive HNE-1 cells, and the plasmid can be successfully expressed. The gene expression in the FR-negative CNE-2 cells may be caused by the PEI cationic polymer 40.

#### Iron in the cell

The endocytosis of MNPS into HNE-1 cells and CNE-2 cells was detected by Prussian blue stain, and the MNPs manifested as blue speckles 41. Figure 3D shows that FR-positive HNE-1 cells absorbed more MNPs than CNE-2 cells at the same density. If the FRs on the HNE-1 cells were closed, there was no obvious absorption of MNPs in HNE-1 cells.

#### RT-PCR and Western blot

To further determine the transfection of the FA-MNP/CDDP/TFPI-2 complexes into HNE-1 cells, we performed RT-PCR and Western blot analysis to detect the mRNA and protein expression of TFPI-2. As shown in Figure 3E(a), the CNE-2 and HNE-1 control groups contained only the TFPI-2 plasmid, and they were not significantly different. After treatment with the FA-MNP/CDDP/TFPI-2 complexes, the TFPI-2 mRNA expression level in the HNE-1 group increased compared with that of the CNE-2 group. This increase in the TFPI-2 mRNA level was accompanied by increased TFPI-2 protein expression (Figure 3E(b)) as determined by Western blot analysis of the TFPI-2 protein in the cell lysates 48 h after transfection.

#### Cell toxicity

**Figure 4:**
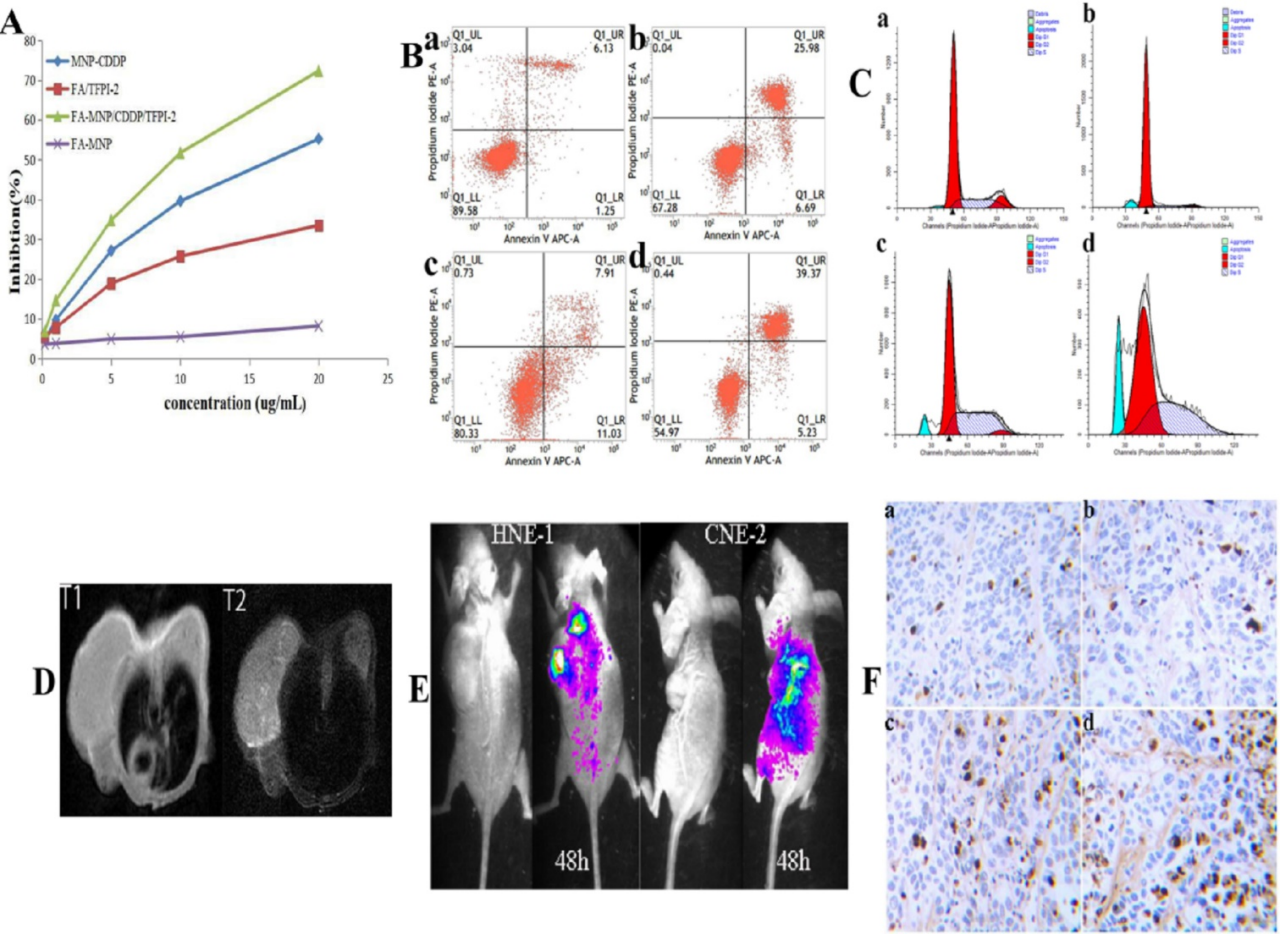
** (A)** CCK-8 results of FA-MNP MNP-CDDP FA/TFPI-2 and FA-MNP/CDDP/TFPI-2 at different concentrations on HEN-1 cells. **(B)** Apoptosis assay (a: FA-MNP group; b: MNP-CDDP group; c: FA-TFPI-2 group; d: FA-MNP/CDDP/TFPI-2 group). **(C)** Cell cycle analysis (a: FA-MNP group; b: MNP-CDDP group; c: FA-TFPI-2 group; d: FA-MNP/CDDP/TFPI-2 group). **(D)** *In vivo* MRI nasopharyngeal carcinoma-bearing nude mice [HNE-1 (right tumor) CNE-2 (left tumor)] (T1: T1-weighted; T2: T2-weighted). **(E)** *In vivo* imaging nasopharyngeal carcinoma-bearing nude mice (Left: HNE-1 nude mouse models; Right: CNE-2 nude mouse models, 48 h: administrated with FA-MNP/CDDP/TFPI-2 for 48 h). **(F)** Tunel staining of tumor tissue sections obtained from mice models 28 days after the final treatment. a: FA-MNP groups; b: FA-TFPI-2 group; c: MNP-CDDP group; d: FA-MNP/CDDP/TFPI-2 group. Positive signals in TUNEL staining were developed by DAB. Cell nuclei were counterstained with hematoxylin. Sepia in Tunel staining indicated apoptosis or necrosis, respectively.

To confirm the cellular inhibition effects of the complexes containing CDDP and TFPI-2, we evaluated their cytotoxic effects by using a CCK-8 assay. Figure 4A shows that the FA-MNPs were nontoxic at the concentration used in this assay, whereas samples containing CDDP or TFPI-2 showed evident cytotoxicity. The cell viabilities of the samples that codelivered CDDP and TFPI-2 were further reduced. The effective inhibition produced by the codelivery to HNE-1 cells may be attributed to the released CDDP and TFPI-2, which can inhibit DNA replication. On the basis of these results, we concluded that codelivery may be an effective tumor therapy method.

#### Apoptosis assay

The control and FA-MNP carrier groups were not significantly different (*p*>0.05). The apoptosis rate of the FA-MNP/CDDP/TFPI-2 group was 44.6%, which was higher than that of the MNP-CDDP group (final concentration CDDP of 4.00 µg/mL) and the FA-TFPI-2 group (gene concentration of 4 µg/mL) (Figure 4B).

#### Cell cycle

The cell cycle was analyzed by flow cytometry to gain further insights into the antiproliferative effects of these nanocomplexes 42. The flow cytometry results showed that the proportion of cells in the G1 phase in the MNP-CDDP group significantly increased and the proportion of cells in the S and G2 phases decreased compared with those in the FA-MNP group. Cellular arrest was further observed in the S and G2 phases of the cell cycle 43. The number of cells in the G2 phase in the FA-TFPI-2 group significantly decreased, and the effects on the cell cycle from TFPI-2 mainly occur in the G2 phase. The cells transfected with FA-TFPI-2 showed the highest increase in the population of cells in the S phase(from 22.64% in the control group to 45.38%), where the treated cells in the MNP-CDDP and FA-MNP/CDDP/TFPI-2 groups exhibited 10.6% and 30% increases, respectively (Figure 4C). Considering these results, we concluded that cell apoptosis in the FA-MNP/CDDP/TFPI-2 group increased and that the cells were blocked in the S and G2 phases.

### VIVO

#### MRI of tumor model

MRI was performed to evaluate the FA-targeting effects of FA-MNP/CDDP/TFPI-2 in the CNE-2 and HNE-1 tumor models. Figure 4D shows that the signal of the HNE-1 tumors was similar to that of the CNE-2 tumors in T1 48 h after FA-MNP/CDDP/TFPI-2 was intravenously administered into the mouse tails. Conversely, the signal of HNE-1 tumors was significantly stronger than that of CNE-2 tumors in T2 44. These results indicated that FA-MNP/CDDP/TFPI-2 exhibited FA-targeting effects.

#### *In vivo* fluorescence imaging

The *in vivo* gene expression capability of FA-MNP/CDDP/TFPI-2 was verified using a Caliper IVIS Lumina II imaging system 45. Figure 4E shows that the CNE-2 and HNE-1 nude mouse models expressed GFP in the abdomen 48 h after FA-MNP/CDDP/TFPI-2 was intravenously administered into the mouse tails. In addition, the HNE-1 tumors exhibited a strong GFP signal, whereas the CNE-2 tumors hardly expressed GFP. This finding indicated that the TFPI-2 plasmid could be delivered by the FA-MNP carriers and that FA-MNP/CDDP/TFPI-2 could target FR-positive tumors.

#### *In vivo* antitumor effects

Additionally, a TUNEL assay was performed to evaluate the therapeutic effects of FA-MNP/CDDP/TFPI-2 (Figure 4F). Minimal apoptosis was observed in tumor tissues treated with FA-MNPs. For mice treated with MNP-CDDP, no evident apoptotic response was observed, but tumor cell necrosis was more significant than that in the FA-TFPI-2 group. Furthermore, extensive apoptosis and necrosis was detected in FA-MNP/CDDP/TFPI-2-treated tumor tissues after 28 days. Moreover, the extent of the apoptotic response was consistent with the scope of tumor necrosis. These results indicated that FA-MNP/CDDP/TFPI-2 codelivering CDDP and TFPI-2 exhibited a synergistic effect to promote apoptosis and effective antitumor activity.

## Conclusion

Our study demonstrates that FA-MNP/CDDP/TFPI-2, containing both CDDP and TFPI-2, was been successfully synthesized and can efficiently inhibit the growth of nasopharyngeal cancer cells both *in vitro* and *in vivo*. FA-MNP/CDDP/TFPI-2 showed good gene delivery ability *in vitro* and could specifically target FA-positive cells and tumor tissue, thus effectively treating FR-positive NPC. For the codelivery assay, FA-MNP/CDDP/TFPI-2 induced more significant apoptosis than CDDP or TFPI-2 alone and exhibited lower toxicity than CDDP alone in HNE-1 cells. In conclusion, this approach provides proof for the use of molecularly targeted technology for the effective and targeted killing of nasopharyngeal cancer cells. Additional studies are required to further affirm the improved therapeutic efficacy of this nasopharyngeal cancer treatment approach. Furthermore, the MNP carrier in FA-MNP/CDDP/TFPI-2 endows it with potential for use in magnetic hyperthermia and magnetic targeted therapy.

## Supplementary Material

Supplementary figures.
