# Supplementary material for: Preparation of FA-targeted magnetic nanocomposites co-loading TFPI-2 plasmid and cis-platinum and its targeted therapy effects on nasopharyngeal carcinoma
Source: Int J Med Sci. 2021 Apr 9;18(11):2355–65. doi: 10.7150/ijms.52643 (PMC8100641; doi:10.7150/ijms.52643)

Preparation of FA-targeted magnetic nanocomposites co-loading TFPI-2 plasmid and cis-platinum and its targeted therapy effects on nasopharyngeal carcinoma

Fang Liu[a&], Bojie Chen[b&],WeifengChen[a],Shuaijun Chen[c],Dong Ma[d],Minqiang Xie[*c]

[a] Department of Otorhinolaryngology Head and Neck Surgery, First Affiliated Hospital of Gannan Medical University,Ganzhou, 341000, China

[b] Department of Joint Surgery, The Affiliated Ganzhou Hospital of Nanchang University, Ganzhou, 341000, China

[c]Department of Otolaryngology, Zhujiang Hospital, Southern Medical University, Guangzhou 510282, China

[d]Key Laboratory of Biomaterials of Guangdong Higher Education Institutes, Department of Biomedical Engineering,Jinan University, Guangzhou, 510632, China

1.TFPI-2 Plasmid construction

We used pEGFP-C1 plasmid as carrier expressing TFPI-2 constructed by GenePharma Co.,Ltd (Suzhou, China), (Homo sapiens tissue factor pathway inhibitor 2 (TFPI-2), transcript variant 1. NM_006528.3 CDS:314..1021).

2. FTIR analyze MPEG-COOH

Figure 1S gives the FTIR spectra of MPEG-COOH and MPEG. As seen, the spectrum of MPEG-COOH did show the characteristic absorption bands of the carboxyl group at 1700 cm^{-1},but MPEG did not exhibit the main characteristic bands of carboxyl. This result confirm that the hydroxy of MPEG changed to carboxyl, and MPEG-COOH have successfully synthesized.

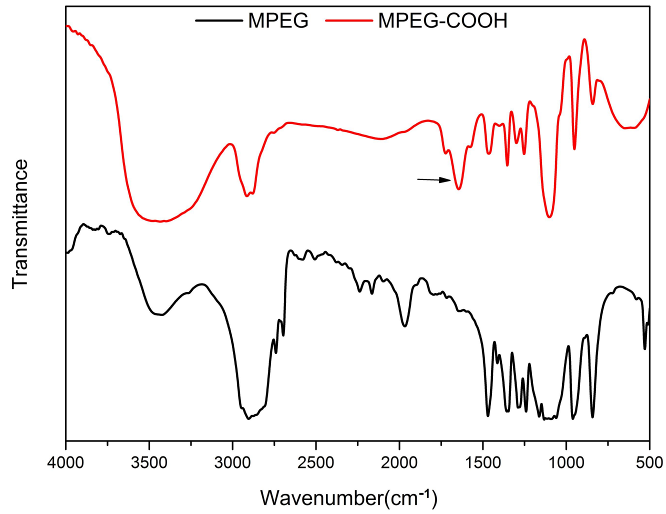

Figure 1S FT-IR of MPEG-COOH

3.**Particle sizes and potential Zeta of FA- MPEG-PEI and MNP**

Figure 2S shown the particle sizes and potential Zeta of FA- MPEG-PEI and MNP, laser particle detector tested FA- MPEG-PEI particle size was 21.6 nm, Zeta potential was +9.42 mV; MNP particle size was 163.0nm, Zeta potential was -26.2 mV. FA-MPEG-PEI Zeta potential diagram appears two peaks at +10.4 mV and -7.51 mV respectively, indicating FA located at the surfaces of FA- MPEG-PEI. Therefore we can deduce that FA appears at the microsphere surface of microsphere mixtures, which play a molecule targeting function. After mixed, FA-MNP nanoparticles of diameter were 151 nm and Zeta potential was +15. 4mV,which showed in the text.

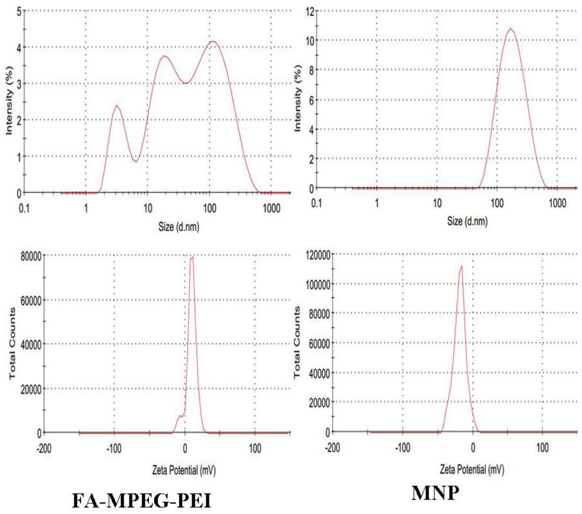

Figure 2S Particle sizes and zeta potentials of FA-MPEG-PEI and MNP

4. **Tumor volume of various groups**

The vivo tumor growth curve was showed in **Figure 3**S(A) and the tumor volume (experimental endpoint) was showed in **Figure 3**S(B). In vivo tumor volume monitored weekly after intravenous injection of different group drugs(a,FA-MNP groups;b:FA-TFPI-2 group;c: MNP-CDDP group; d: FA-MNP/CDDP/TFPI-2 group). The treatments with FA-MNP show tumor volume increases slowly.Compared to the treatments with FA-TFPI-2 and MNP-CDDP, FA-MNP/CDDP/TFPI-2 group show considerable effect(**Figure 3**S(A)) and substantially suppressed tumor growth(**Figure 3**S(B)). At the same drug dosage (CDDP 3 mg/kg), the FA-MNP/CDDP/TFPI-2 anti-tumor potency of was higher than MNP-CDDP.

Figure 3S The vivo tumor growth curve and the tumor volume

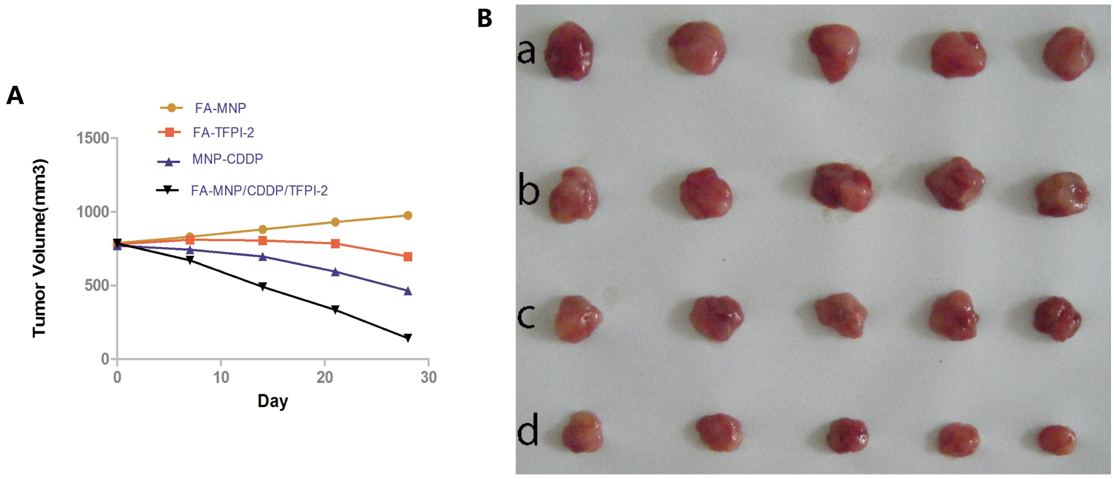

Supplement: Supplementary file 1 — Supplementary figures. [file ijmsv18p2355s1.pdf]
